# Deciphering α-L-Fucosidase Activity Contribution in Human and Mouse: Tissue α-L-Fucosidase FUCA1 Meets Plasma α-L-Fucosidase FUCA2

**DOI:** 10.3390/cells14171355

**Published:** 2025-08-30

**Authors:** Hannah Bäumges, Svenja Jelinek, Heike Lange, Sandra Markmann, Emanuela Capriotti, Jan Anwar Häusser, Mai-Britt Ilse, Thomas Braulke, Torben Lübke

**Affiliations:** 1 Department of Chemistry, Biochemistry III, Bielefeld University, 33615 Bielefeld, Germany; hannah.baeumges@uni-bielefeld.de (H.B.); svenja.jelinek@uni-bielefeld.de (S.J.); jan.haeusser@uni-bielefeld.de (J.A.H.); mai-britt.ilse@uni-bielefeld.de (M.-B.I.); 2 Institute of Osteology & Biomechanics, Cell Biology of Rare Diseases, University Medical Center Hamburg-Eppendorf, 20246 Hamburg, Germany; san.markmann@gmail.com (S.M.); e.capriotti@uke.de (E.C.); braulke@uke.de (T.B.)

**Keywords:** α-L-fucosidase 1, FUCA1, α-L-fucosidase 2, FUCA2, lysosomes, lysosomal storage disease, fucosidosis

## Abstract

Fucose-containing glycoproteins and glycolipids broadly occur in humans as well as in many other species and are essential for a wide range of physiological processes, such as cell adhesion, fertilization, and tumor development. In humans, the cellular degradation of various fucosylated glycoconjugates depends on the *FUCA1*-encoded lysosomal tissue α-L-fucosidase (FUCA1). The crucial role of FUCA1 is reflected by the severe lysosomal storage disease fucosidosis, which causes a massive accumulation of fucosylated glycans, glycolipids, and α(1,6)-fucosylated glycoasparagines. Therefore, it is reasonable to assume that FUCA1 is predominantly responsible for the degradation of fucosylated glycoconjugates, although a second, functionally uncharacterized α-L-fucosidase, the plasma α-L-fucosidase (FUCA2), is known. To investigate the impact of both fucosidases in more detail, we generated two different monoclonal antibodies as useful tools for the detection of human and murine FUCA1 and utilized a FUCA2-specific antibody to demonstrate that FUCA2 is a bona fide lysosomal protein that is sorted in a mannose 6-phosphate (M6P)-dependent manner. We then compared FUCA1 and FUCA2 upon ectopic expression and evaluated their enzyme activity profiles under various conditions. Untagged and differently tagged versions of FUCA1 exhibited α-L-fucosidase activity, while various FUCA2 derivatives, even after affinity purification, did not show any fucosidase activity against commonly used pseudo-substrates. Our findings suggest that FUCA1 and not FUCA2 is exclusively responsible for the lysosomal de-fucosylation of glycoconjugates.

## 1. Introduction

Fucosidases are hydrolytic enzymes that are critical for the degradation of fucosylated glycoconjugates, including fucosylated N- and O-glycans as well as fucosylated glycolipids [1]. In humans, fucosylation plays an important role in numerous cellular and (patho)physiological processes, such as cell adhesion, fertilization, tissue development and homeostasis, inflammatory processes, angiogenesis, cancer progression, and immune cell development [2,3,4]. Enzyme-mediated de-fucosylation mainly depends on the lysosomal tissue α-L-fucosidase 1 (FUCA1; EC 3.2.1.51; CAZy family GH29), which is capable of cleaving all known α-L-fucose linkages, including α(1,2)-, α(1,3)-, α(1,4)-, and α(1,6)-fucosylated glycoconjugates [5,6]. The human *FUCA1* gene, located on chromosome 1, comprises eight exons encoding a precursor protein of 466 amino acids (NCBI NP_000138). Following the co-translational cleavage of the 27-amino-acid signal sequence, the approx. 50 kDa mature enzyme is generated, which is co-translationally modified at three asparagine residues (N241, N268, and N382) with partially sialylated [7,8] and mannose 6-phosphorylated oligosaccharides [9]. Mannose 6-phosphate (M6P) serves as a specific recognition marker for efficient lysosomal targeting [10] and is formed on almost all of the approx. 70 luminal and soluble lysosomal proteins by the enzymatic action of the Golgi-resident GlcNAc-1-phosphotransferase (GNPTAB) complex [10]. N-glycan sialylation and mannose 6-phosphorylation also contribute to the occurrence of various isoelectric forms of FUCA1 [11,12]. Oligomerization of FUCA1 was suggested long ago [13]. A recent study on human FUCA1 using cryo-electron microscopy (Cryo-EM) revealed a homotetrameric quaternary structure and functionally highlighted aspartate residues as the catalytic nucleophile and acid/base residue, both of which are critical for proper fucosidase activity [14]. The catabolic impact and clinical relevance of FUCA1 function are particularly evident in the ultra-rare and fatal lysosomal storage disease fucosidosis. This inherited disorder is biochemically characterized by the loss of FUCA1 activity, which leads to the progressive accumulation of fucosylated glycoconjugates, mainly the glycoasparagine Fuc(α1,6)GlcNAcß1-Asn, in many tissues and cell types [6,15]. Clinically, patients manifest a wide range of different symptoms, such as progressive neurological deterioration and intellectual disability, seizures, growth retardation, dysostosis multiplex, and angiokeratoma corporis diffusum [6]. Various models of the human disease have been established in mice [16,17] or occur naturally in cats [18] and dogs [19]. FUCA1-deficient dogs have been used for enzyme replacement therapy [20]. Interestingly, neither fucosidosis patients nor animal models of this disease show quantifiable amounts of residual fucosidase activity, although a second lysosomal fucosidase, initially named plasma α-L-fucosidase (FUCA2; NCBI NP_114409), has been reported [21,22]. The human *FUCA2* gene is located on chromosome 6, consists of seven exons, and encodes a 467-amino-acid protein with a 28-amino-acid signal peptide and three putative N-glycosylation sites (N171, N239, and N377), of which the glycan at position N377 is M6P modified, indicating a lysosomal localization [23]. Both human fucosidases belong to the glycoside hydrolase (GH) family 29 [24] and show an amino acid identity of 55% and an amino acid similarity of 70%. However, to date, only one experimental study has reported that the enzymatic activity of FUCA2 may promote the expression of fucosylated Lewis x structures in *Helicobacter pylori*, which are thought to be necessary for the interaction with host gastric mucosa cells [25]. Interestingly, FUCA2 was consistently identified in M6P-glycoproteome analyses, suggesting a role as a putative lysosome-resident glycoprotein rather than an exclusively secreted protein [9,26]. Finally, subcellular localization studies based on immunofluorescence microscopy analyses of FUCA2-mCherry fusion proteins also indicated a lysosomal localization of FUCA2 [27].

In this study, we established and characterized self-generated FUCA1-specific monoclonal antibodies and demonstrated their suitability, and that of a commercially available FUCA2 antibody, for functionality in western blotting and immunofluorescence microscopy. We have shown for the first time that, in addition to FUCA1, FUCA2 is also a genuine lysosomal protein that is sorted into lysosomes in an M6P-dependent manner. Furthermore, comparative expression analyses of both α-L-fucosidases revealed that only FUCA1-containing cell extracts, but not FUCA2-containing cell extracts or affinity-purified FUCA2, exhibited fucosidase activity towards the commonly used fucosidase pseudo-substrates 4-methylumbelliferyl-α-L-fucopyranoside (4-MUF) and p-nitrophenyl-α-L-fucopyranoside (pNPF). The present data argue against the significant function of FUCA2 in the bulk de-fucosylation of oligosaccharides in humans.

## 2. Materials and Methods

### 2.1. Bioinformatics

**Figure 1:**
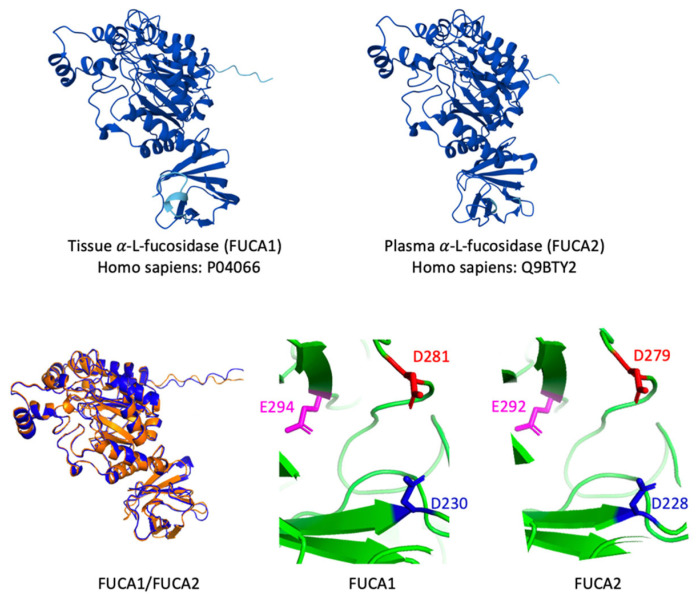
Structural comparison of human *FUCA1*-encoded tissue α-L-fucosidase and human *FUCA2*-encoded plasma α-L-fucosidase from the AlphaFold3 structural database. The dark blue colored structural elements indicate a very high AlphaFold confidence (pLDDT > 90); light blue indicates high confidence (pLDDT > 70). (**upper panel**; both proteins shown without signal peptide). Superimposition of human FUCA1 (orange) and human FUCA2 (blue) was performed in PyMOL (**lower panel**). The catalytic cores of both proteins are very similar with regard to critical residues. The nucleophilic aspartates D230 in FUCA1 and D228 in FUCA2 are shown in blue; the catalytic acid/ base aspartates D281 and D279 are shown in red. The glutamates E294 in FUCA1 and E292 in FUCA2 stabilize the catalytic centers. (pLDDT – predicted local distance difference test).

Human *FUCA1* (NM_000147) encodes human FUCA1 (NP_000138), and human *FUCA2* (NM_032020) encodes human FUCA2 (NP_114409). Murine *Fuca1* (NM_024243) and *Fuca2* (NM_001330198) encode mouse Fuca1 (NP_077205; 452 aa) and Fuca2 (NP_001317127; 461 aa), respectively. The structures and maps of FUCA1 and FUCA2 shown in Figure 1 were prepared using AlphaFold Protein Structure Database and PyMol with PDB files P04066 for FUCA1 and Q9BTY2 for FUCA2, respectively.

### 2.2. Cell Culture

Cell lines (HT1080, HeLa, HEK293FT, Neu2A; all obtained from the American Type Culture Collection (ATCC, Manassas, VA, USA)) were grown adherently in DMEM (Gibco, Life Technologies GmbH, Darmstadt, Germany) medium containing 10% FBS (fetal bovine serum; PAN, Aidenbach, Germany), 1% penicillin/streptomycin (Bio&Sell, Feucht, Germany) and 1% L-glutamine (Bio&Sell) at 37 °C, 5% CO_2_ and 99% humidity. To obtain stably expressing cell lines, cells were selected with 400 µg/mL hygromycin B (Roth, Karlsruhe, Germany). CHO-K1 cells were additionally supplemented with L-proline (40 µg/mL final concentration; Sigma, Schnelldorf, Germany). Wild-type (WT) and *GNPTAB* knockout (KO) haploid (HAP1) cells were obtained from Horizon Discovery (Waterbeach, UK) and grown in Iscove’s modified Dulbecco’s medium (IMDM, Gibco, Life technologies GmbH, Darmstadt, Germany) supplemented with 10% heat-inactivated FBS, 2 mM L-glutamine, and 1% penicillin/streptomycin [28].

### 2.3. Cloning of Fucosidase Expression Constructs

Coding sequences for human *FUCA1*, mouse *Fuca1*, human *FUCA2,* and mouse *Fuca2* were obtained from total RNA by reverse transcription followed by add-on PCR using specific primer pairs. All sequences were subcloned into the mammalian expression vector pcDNA3.1Hygro^(+)^ (Thermo Fisher Scientific, Dreieich, Germany) using the NheI and XhoI restriction sites, as all add-on forward primers included a NheI site and all add-on reverse primers included a XhoI site. The following tag sequences were used either N-terminally (HA-tag) or C-terminally (H6- and Flag-tag): H6-tag: GRGSHHHHHHG encoded by *GGA AGA GGA TCG CAT CAC CAT CAC CAT CAC GGA*; Flag-tag: DYKDDDDK encoded by *GAC TAC AAA GAC GAT GAC GAC AAG*; HA-tag: YPYDVPDYA encoded by *TAC CCA TAC GAT GTT CCA GAT TAC GCT*.

Finally, the following constructs were used in this study:

For FUCA1: pcDNA3.1Hygro^(+)^-mock; pcDNA3.1Hygro^(+)^-*FUCA1*; pcDNA3.1Hygro^(+)^-*FUCA1*-H6; pcDNA3.1Hygro^(+)^-*FUCA1*-Flag; pcDNA3.1Hygro^(+)^-HA-*FUCA1*; pcDNA3.1Hygro^(+)^-*Fuca1*;

For FUCA2: pcDNA3.1Hygro^(+)^-*FUCA2*; pcDNA3.1Hygro^(+)^-*FUCA2*-H6; pcDNA3.1Hygro^(+)^-HA-*FUCA2*; pcDNA3.1Hygro^(+)^-HA-*FUCA2*-H6; pcDNA3.1Hygro^(+)^-*Fuca2*-H6.

Different tags and tag positions were used for the *FUCA2* constructs in order to avoid interference of the tag with enzymatic function. The integrity of all constructs was confirmed by Sanger sequencing.

### 2.4. Generation of Fucosidase-Specific Monoclonal Antibodies

*Fuca1*-knockout mice (*Fuca1* KO; [16]) were immunized by ballistic injection of coated gold particles carrying a mouse tissue α-L-fucosidase 1-encoding construct (pcDNA3.1Hygro^(+)^-*Fuca1;* untagged), slightly modified as described previously [29]. Additionally, immune response in mice was boosted by using purified H6-tagged human α-L-fucosidase 1 (FUCA1-H6) derived from CHO-K1 cells, resulting in a FUCA1-positive antiserum from mouse #4. To obtain monoclonal antibody-producing hybridoma cells, spleen-derived B-lymphocytes were fused with myeloma cells, selected on HAT medium, and single-cell-derived clones were obtained. Supernatants from these hybridomas were tested by western blot analyses to detect human and mouse recombinant tissue α-L-fucosidase and plasma α-L-fucosidase, respectively. Two FUCA1-detecting hybridoma clones, A112 and A180, were established.

### 2.5. RNA Isolation, cDNA Synthesis, and Real-Time PCR

Mouse RNA was isolated from up to 20 mg of fresh tissue from wild-type and *Fuca1*-KO mice according to the manufacturer’s recommendation (innuPREP RNA Mini Kit 2.0, IST Innuscreen, Berlin, Germany). cDNA was subsequently synthesized using the FastGene Scriptase II cDNA Kit (5× ReadyMix OdT, NIPPON Genetics, Düren, Germany). qPCR analysis was performed on a StepOnePlus real-time PCR cycler (Thermo Fisher Scientific, Dreieich, Germany) using the qPCRBIO SyGreen Mix Hi-Rox (PCR Biosystems, London, UK) and the following primers:

*Fuca1*-F 5′-GAA CAT ACG CTA CGG CCT CT-3′; *Fuca1*-R 5′-GGT CAG GCT TGT AGC TGT TA-3′

*Fuca2*-F 5′-CAC TCC GGA TGT GTG GTA CA-3′; *Fuca2*-R 5′-CCA ATG GCC CAG CAG TTC TA-3′; *Ppia*-F 5′-CAG GTC CAT CTA CGG AGA GA-3′; *Ppia*-R 5′-CAT CCA GCC ATT CAG TCT TG-3′. *Ppia* (peptidylprolyl isomerase A) expression was used for normalization [30].

### 2.6. Transient Transfection, Preparation of Cleared Cell Lysates, and Immunoblotting

Fucosidase expression constructs (all in mammalian expression vector pcDNA3.1Hygro^(+)^) were transiently expressed in Neu2A, HT1080, HeLa, or HEK293FT cells using a polyethylenimine (PEI) transfection protocol as described previously [31]. Cells were harvested 24 h after transfection and lysed in TRIS-buffered saline (TBS; pH 7.4) containing 0.5% TX100 for 20 min and additionally by sonication on ice (3 × 10 s). Cleared lysates were obtained by centrifugation (13,000× *g*, 4 °C, 10 min). Protein concentration was determined by detergent compatible (DC) assay (BioRad, Feldkirchen, Germany). Aliquots of lysates were analyzed by immunoblotting on polyvinylidene difluoride (PVDF) membrane using the following antibodies: hybridoma supernatants containing FUCA1 and Fuca1 antibodies (A112 and A180; 1:500), FUCA2 (15157-1-AP, polyclonal rabbit; 1:1000; Proteintech, Planegg-Martinsried, Germany), H6-tag (anti-RGS-(H)_4_; 1:500; Qiagen, Hilden, Germany), Flag-tag (anti-Flag M2; 1:500; Cell Signaling Technology, Leiden, The Netherlands), HA-tag (51064-2-AP; 1:500; Proteintech, Planegg-Martinsried, Germany), and GAPDH (sc-25778, FL-335; 1:500; Santa Cruz Biotechnology, Heidelberg, Germany; ). Primary antibodies and HRP-conjugated secondary antibodies (1:5000; Dianova, Hamburg, Germany) were diluted in TBS with 0.1% (*w*/*v*) Tween20 and 5% (*w*/*v*) skimmed milk powder. Signals were visualized using the SuperSignal West Pico Plus ECL detection system (Life Technologies, Darmstadt, Germany).

### 2.7. Deglycosylation of N-Glycosylated Proteins

Cell lysates (40 µg total protein) were treated with peptide N-glycosidase F (PNGase F; V483A, Promega, Walldorf, Germany) as recommended by the manufacturer.

### 2.8. α-L-Fucosidase Activity Assays

α-L-fucosidase activity was measured using 4-methylumbelliferyl-α-L-fucopyranoside (4-MUF; Carbosynth, Compton, UK) or p-nitrophenyl-α-L-fucopyranoside (pNPF) as indicated. Cleared cell lysates (10 µL containing 2–3 µg/µL total protein) were incubated with either 150 μL of 0.75 mM 4-MUF in 0.1 M sodium citrate buffer (pH 5.5) containing 0.2% BSA, or 100 μL of 2 mM pNPF in 0.1 M sodium citrate buffer (pH 5.5) containing 0.2% BSA. The samples were incubated at 37 °C, and the reactions were stopped by adding 150 μL of 1 M sodium carbonate (pH 10.4) for the 4-MUF assay, or 800 µL of 0.4 M glycine in NaOH (pH 10.4) for the pNPF assay. The amount of liberated 4-MU was determined by fluorescence measurement (excitation: 360 nm; emission: 465 nm) using a Spark 10M microplate reader (Tecan, Männedorf, Switzerland) and calculated using a standard curve (0–10 nmol 4-MU). If necessary, lysates were diluted 1:10 or 1:100 to remain within the linear range. To calculate the α-L-fucosidase activity, the amount of liberated pNP was determined in a 96-well plate (300 µL total volume) by measuring the absorbance at 405 nm using a Spark 10M microplate reader. To analyze the pH dependency of α-L-fucosidase activity, McIlvaine buffer was used instead of 0.1 M sodium citrate and adjusted to the indicated pH values.

### 2.9. Purification of Tagged and Untagged FUCA1 and FUCA2

HT1080 cells were PEI-transfected with either the H6-tagged FUCA1-encoding or the H6-tagged FUCA2-encoding construct (each in pcDNA3.1Hygro^(+)^), and subsequently selected with 600 µg/mL hygromycin for 8–10 days. Hygromycin-resistant cells were expanded and switched to conditioned, FCS-free DMEM instead of 10% FCS. The supernatants containing the H6-tagged fucosidase were collected three times at 48 h intervals and subjected to Ni-NTA affinity purification. Briefly, fucosidase-containing conditioned medium was incubated for one hour at 4 °C with 2 mL Ni-NTA suspension (50% slurry) with gentle agitation. Washing was performed with 5 mL 20 mM imidazole in 20 mM TRIS/HCl (pH 7.4), containing 500 mM NaCl, and elution was performed in a single step using 1 mL 500 mM imidazole in 20 mM TRIS/HCl (pH 7.4), containing 500 mM NaCl. For further applications, purified proteins were concentrated 5-fold and dialyzed against appropriate buffers such as McIlvaine buffer using 15 mL centricons with an MWCO of 10 kDa, resulting in 200 µL fractions. Thus, 10 µL of the final affinity-purified FUCA1- and FUCA2-fractions corresponds to the equivalent of 50 mL of the original conditioned medium used for purification. Untagged FUCA1 was stably expressed in adherent CHO-K1 cells and collected from FCS-free medium. Protein from the conditioned medium was precipitated by adding 0.5 g/mL ammonium sulfate. After reconstitution and dialysis in strong cation binding buffer (50 mM NaOAc, pH 5.0), untagged FUCA1 was eluted in a gradient from 10 mM to 1M NaCl from a HiTrap strong cation exchange (SCX) SP HP column (Cytiva, Freiburg, Germany) (elution buffer: 50 mM NaOAc, pH 5.0, 1 M NaCl) in a single peak at 450 mM NaCl, as measured by the pNPF fucosidase activity assay. For further applications, purified proteins were concentrated and dialyzed against appropriate buffers using 15 mL centricons with a MWCO of 10 kDa. Affinity-purified FUCA1-H6 or FUCA2-H6 were separated by SDS-PAGE and detected by the sensitive blue silver colloidal Coomassie G-250 staining procedure [32].

### 2.10. Size-Exclusion Chromatography

Ni-NTA affinity-purified FUCA1-H6, SCX-purified untagged FUCA1, and lysosome-enriched fractions from tyloxapol-treated mouse liver, so-called tritosomes [33], as well as Ni-NTA affinity-purified FUCA2-H6, were buffered in McIlvaine buffer (pH 4.6) or PBS (pH 7.4) and analyzed by size exclusion chromatography using a Superdex 200 Increase 10/300 GL column (Cytiva Europe GmbH, Freiburg, Germany) with a flow rate of 0.3 mL/min. Fractions of 250 µL containing FUCA1(-H6) and tritosomes were collected from 8 mL to 17 mL retention volume and analyzed by the 4-MUF fucosidase activity assay.

For FUCA2-H6 analysis, the corresponding fractions (250 µL) were precipitated with TCA (10% final concentration) on ice in order to obtain sufficient protein for western blot analysis. TCA-treated samples were centrifuged at 4 °C, 15,000× *g* for 20 min, and the resulting protein pellets were washed twice with ice-cold acetone, dried at room temperature (RT), and resuspended in Laemmli buffer for SDS-PAGE.

### 2.11. Indirect Immunofluorescence Microscopy

HT1080 cells were seeded on coverslips, transfected with human FUCA1-, FUCA2- or mouse Fuca1-encoding constructs (see above) and cultured for 24 h. Cells were fixed and permeabilized for 30 s at RT using 100% methanol pre-chilled to −20 °C. They were blocked with 1% BSA in PBS for 30 min and incubated with primary antibodies against FUCA1/Fuca1 (A180 or A112; each 1:25), FUCA2 (15157-1-AP; 1:400), and LAMP1 (Abcam (Cambridge, UK), Ab24170; 1:200; or H4A3, Development Studies Hybridoma Bank; 1:25) for 1 h at RT. All antibodies were diluted in PBS with 1% BSA. Goat anti-mouse and goat anti-rabbit secondary antibodies conjugated with Alexa Fluor 488 or Alexa Fluor 647 (Life Technologies GmbH) were diluted 1:1000 in PBS with 1% goat serum and incubated for 1 h at RT. Hoechst staining (1:500 in H_2_O) was performed to visualize nuclei. Coverslips were mounted with Mowiol. Images were acquired using a Zeiss LSM700 confocal microscope (Carl Zeiss, Oberkochen, Germany) with a 100 × oil immersion objective.

### 2.12. Lysosome-Enriched Fractions from Mouse Liver (Tritosomes)

Lysosome-enriched fractions from the livers of 6-month-old mice were isolated following intravenous injection of the detergent tyloxapol (0.75 mg/g body weight; Sigma, Schnelldorf, Germany) by a combination of differential and isopycnic centrifugation steps, as described previously [33].

### 2.13. Enrichment and Analysis of Lysosome-Enriched Fractions from HAP1 Cells

The subcellular fractionation procedure to enrich lysosomal fractions and preparation of conditioned media from HAP1 cells have been described previously [28]. Briefly, serum-free media (OptiMEM, ThermoFisher, Life Technologies GmbH) conditioned for 24 h by WT and GNPTAB-KO HAP1 cells were collected, centrifuged, and concentrated 10-fold using Amicon© Ultra-0.5 mL centrifugal filters (Merck Millipore, Darmstadt, Germany; 10 kDa MWCO). The cells were washed and harvested in 10 mM HEPES (pH 7.4) containing 250 mM sucrose (buffer A), followed by homogenization in buffer A containing 1 mM EDTA using a 7 mL Dounce homogenizer. After centrifugation at 800× *g* and 4 °C for 10 min, the post-nuclear supernatants (PNS) were collected, and the nuclear pellets were re-homogenized and centrifuged as described above. The pooled PNS fractions were centrifuged at 20,000× *g* for 30 min, the supernatants were removed, and the resulting pellets (20K fraction) were resuspended in RIPA buffer (25 mM Tris-HCl, pH 7.6, containing 150 mM NaCl, 1% NP-40, 1% sodium deoxycholate, 0.1% SDS, and protease inhibitors (Roche, Grenzach, Germany)) and further processed for protein determination and western blotting using NuPAGE 4–12% Bis-Tris Protein Gels (Thermo Fisher, Life Technologies GmbH). After blocking with 5% non-fat milk in TBST, the nitrocellulose membranes were incubated overnight at 4 °C with the following antibodies in blocking buffer: rat anti-GNPTAB α-subunit hybridoma supernatant (1:50; [34]), mouse anti-LAMP1 (1:1000; Santa Cruz Biotechnology (Dallas, TX, USA) sc-20011), mouse anti-LAMP2 (2D5; 1:1000; [35]), rabbit anti-cathepsin D (1:1000; Merck, #IM16), mouse anti-FUCA1 A112 hybridoma supernatant (1:500), or rabbit anti-FUCA2 (1:500; Proteintech (Planegg-Martinsried, Germany), 15157-1-AP). Horseradish peroxidase (HRP)-conjugated anti-rabbit IgG (Biozol (Hamburg, Germany), JIM-111-035-003), anti-mouse IgG (Jackson (Cambridge, UK), 115-035-003), or anti-rat IgG (Jackson,112-035-003) were used as secondary antibodies and incubated in blocking buffer for 1h at room temperature at a dilution of 1:5000. Immunoreactive proteins were detected by enhanced chemiluminescence (Clarity western ECL substrate kit; BioRad (Feldkirchen, Germany), 1705060) and imaged using a Chemidoc Imaging system (Bio-Rad).

### 2.14. Animals

Fuca1-knockout mice [36] were kept under standard laboratory conditions in a pathogen-free animal facility at Bielefeld University. All procedures involving mice were performed according to local guidelines and approved by local authorities in accordance with EC regulations.

## 3. Results

### 3.1. Structural Comparison of FUCA1 and FUCA2

The AlphaFold3 Protein Structure Database [37] predicts nearly identical folds with very high confidence scores (pLDDT > 90) for human α-L-fucosidase 1 and 2 (Figure 1, upper panel). Moreover, the positions of enzymatically critical amino acids in the catalytic center of both FUCA1 (D230, D281, E294) and FUCA2 (D228, D279, E292) are very similar, suggesting that the *FUCA2*-encoded plasma α-L-fucosidase might also be an active fucosidase (Figure 1, lower panel). In FUCA1, the aspartates D230 and D281 act as the catalytic nucleophile and the catalytic acid/base residue, respectively. Furthermore, replacement of glutamate E294 in FUCA1 was shown to result in a substantial loss of activity, as this residue stabilizes the catalytic center [14].

### 3.2. Transcript Analysis of Fuca1 and Fuca2 in Wild-Type and Fuca1-KO Mouse Tissues

**Figure 2:**
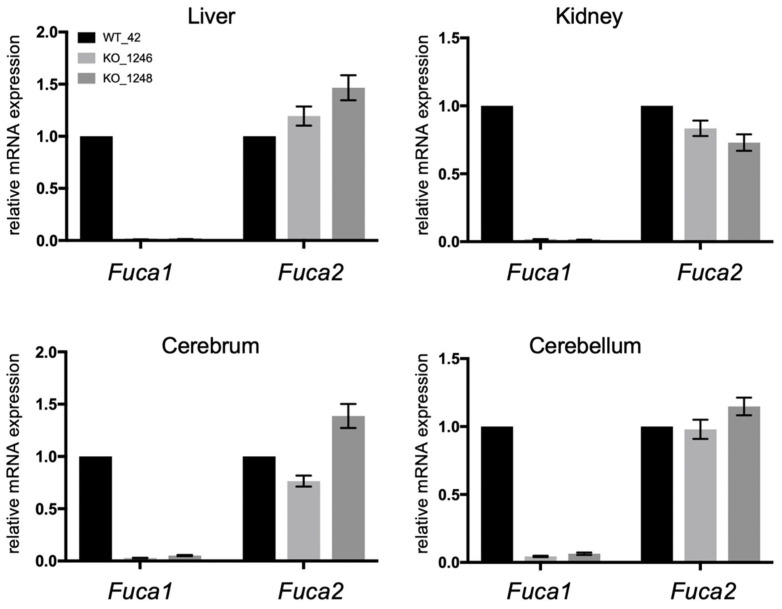
Comparative qPCR (ΔΔCt analysis) of *Fuca1* and *Fuca2* transcript levels in WT and *Fuca1*-KO mice, using *Ppia* as a reference gene. *Fuca1* transcripts were significantly reduced in all knockout samples, while *Fuca2* expression levels remained largely unchanged in the *Fuca1*-knockout background. The relative mRNA expression levels of WT tissues were set to 1.

We wondered whether the loss of *Fuca1* gene expression might be compensated by an increased expression of the *Fuca2* gene. Transcript levels of mouse *Fuca1* and *Fuca2* were determined by quantitative (q)PCR using RNA from various tissues of 6-month-old wild-type (#42) and *Fuca1*-KO mice (#1246, #1248), respectively (Figure 2). In wild-type tissues, *Fuca1* and *Fuca2* were expressed at similar levels in the kidney, cerebrum, and cerebellum, as indicated by comparable cycle threshold (C_t_) values: 21.18 (*Fuca1*) and 20.81 (*Fuca2*) in the kidney; 23.88 (*Fuca1*) and 23.95 (*Fuca2*) in the cerebrum; 23.66 (*Fuca1*) and 23.89 (*Fuca2*) in the cerebellum. Only in the liver was *Fuca1* expression (C_t_ 22.95) higher than that of *Fuca2* (C_t_ 25.90). In *Fuca1*-KO tissues, *Fuca1* transcripts were nearly absent, while *Fuca2* expression levels remained almost unchanged in all tissues, independent of *Fuca1* expression, suggesting that the loss of *Fuca1* has no direct compensatory impact on *Fuca2* expression.

### 3.3. Generation and Evaluation of α-L-Fucosidase-Specific Antibodies

**Figure 3:**
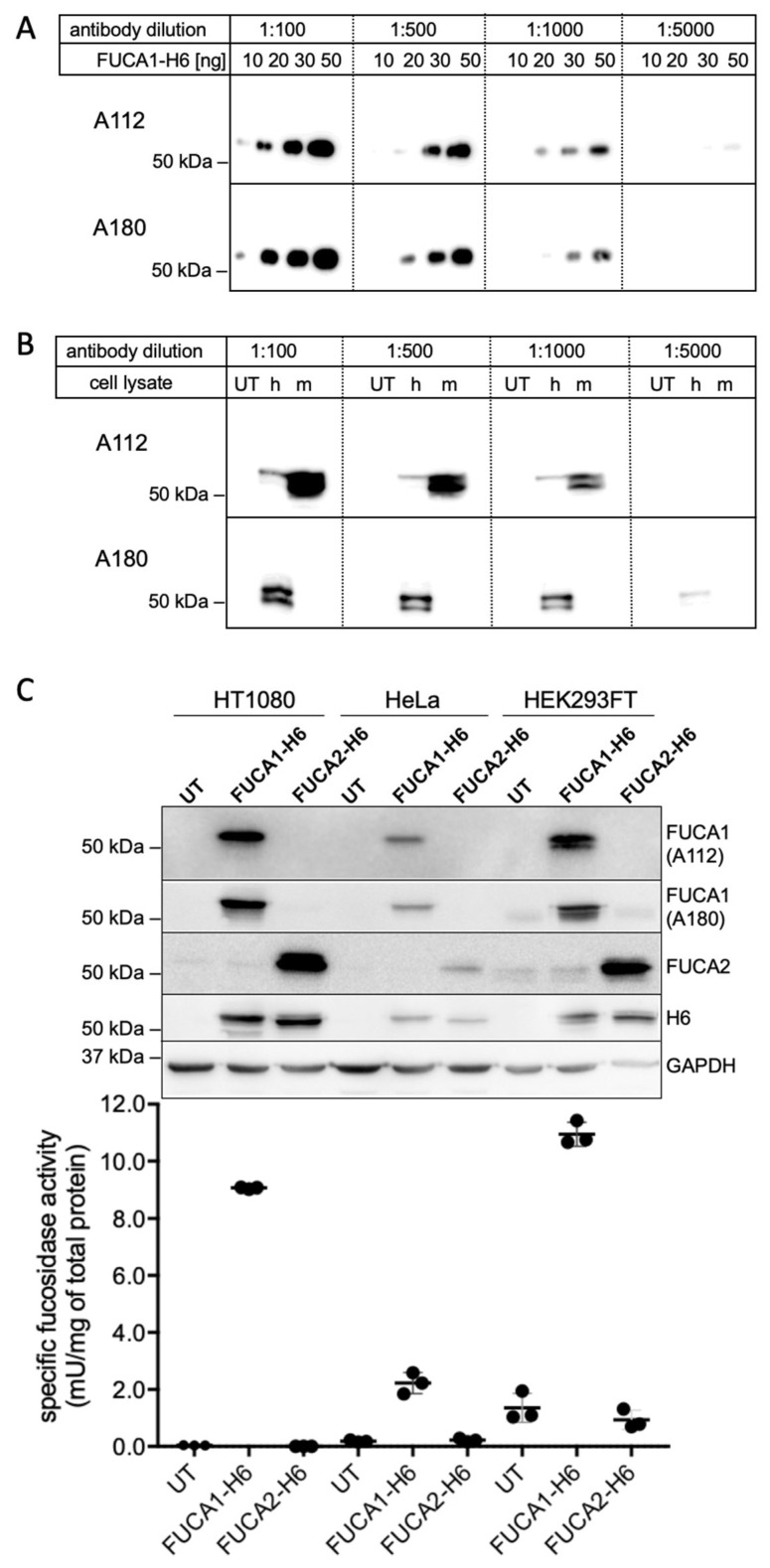
(**A**) Monoclonal antibodies A112 and A180 detected human FUCA1 in a dose-dependent manner. The hybridoma supernatants were analyzed by western blotting using different amounts (10–50 ng) of H6-tagged human fucosidase (FUCA1-H6) and four different dilutions of the hybridoma supernatants ranging from 1:100 to 1:5000. (**B**) Lysates (50 µg protein) from untransfected (UT) and transiently transfected Neu2A cells expressing either untagged human (h) FUCA1 or untagged mouse (m) Fuca1 were blotted and analyzed with the indicated dilutions of the hybridoma supernatants A112 or A180. (**C**) Lysates (25 µg protein) from untransfected (UT) or transiently transfected human HT1080 cells, HeLa cells, and HEK293FT cells expressing FUCA1-H6 or FUCA2-H6 were analyzed by western blotting with the indicated antibodies. Lysates were also tested for specific α-L-fucosidase activity using the pNPF assay (mean ± SD; *n* = 3).

Commercially available antibodies and antisera directed against lysosomal tissue α-L-fucosidase (*FUCA1*-encoded) failed to specifically detect human and mouse recombinant α-L-fucosidase 1. Therefore, we immunized *Fuca1*-KO mice by ballistic injection of coated gold particles carrying an untagged mouse *Fuca1* cDNA construct (pcDNA3.1Hygro(+)-*Fuca1*). The same mice were further boosted with purified H6-tagged human α-L-fucosidase 1 protein (FUCA1-H6), resulting in a mouse serum that sensitively detected recombinant human FUCA1 in a low nanogram range. To obtain a sustainable source for monoclonal antibodies against FUCA1, antibody-producing hybridoma cell lines were established using standard protocols. Two supernatants from the hybridoma cell lines A112 and A180, respectively, showed high sensitivity for recombinant glycosylated human FUCA1 with an apparent molecular mass of 50–55 kDa, even at high dilutions of the supernatants (1:1000) in a dose-dependent manner (Figure 3A).

As the immunization protocol used the murine *Fuca1* cDNA construct for the initial immunization and purified recombinant human FUCA1 for the booster, the resulting hybridomas might recognize both, mouse and human orthologs, which share an 85% sequence identity of the mature proteins without signal sequences (NCBI Protein Blast). We therefore tested cross-reactivity against both orthologs by western blotting using lysates from transfected mouse neuroblastoma Neu2A cells expressing either the human construct (pcDNA3.1Hygro(+)-*FUCA1*) or the murine construct (pcDNA3.1Hygro(+)-*Fuca1*). Lysates from untransfected Neu2a cells (UT) did not show specific signals with either of the two monoclonal antibodies. The A112 antibody preferentially recognized the mouse (m) Fuca1 but also the human (h) FUCA1 (Figure 3B). In contrast, the A180 antibody exclusively detected the human FUCA1 (Figure 3B). Notably, both human FUCA1 and mouse Fuca1 were detected as a 50/55 kDa doublet.

We applied the FUCA1 specific antibodies A112 and A180, as well as a commercially available polyclonal rabbit antibody against FUCA2, to analyze cell lysates derived from the human fibrosarcoma cell line HT1080, HeLa cells and HEK293FT cells after transient transfection with the *FUCA1*-H6 and the corresponding *FUCA2*-H6 construct (Figure 3C). A112 and A180 detected strong FUCA1-specific signals in HT1080 and HEK293FT cells, but much weaker signals in HeLa cells (Figure 3C, upper panel). The FUCA2 antibody detected FUCA2 in HT1080 and HEK293 cells with comparable strong signals, whereas the FUCA2 signal in the HeLa lysate was much weaker. Probing the blot with the H6-tag specific antibody revealed comparable signals for the FUCA1-H6- and the FUCA2-H6-containing lysates of all three human cell lines, confirming that FUCA1 and FUCA2 expression in HeLa cells was less efficient than in HT1080 and HEK293FT cells. Notably, the molecular mass of FUCA2-H6 was slightly lower than that of the FUCA1-H6 variant.

We then tested the same FUCA1-H6- and FUCA2-H6-containing cell lysates for α-L-fucosidase activity using the pNPF substrate assay (Figure 3C, lower panel). As expected from the western blot signals, the FUCA1-containing lysates of HT1080 and HEK293FT cells showed the highest fucosidase activities. FUCA1-H6-containing HeLa cells exhibited only about 20% of the specific α-L-fucosidase activity observed in HT1080 or HEK293FT cells. Most importantly, none of the FUCA2-H6-containing samples showed higher α-L-fucosidase activity than untransfected (UT) cells.

### 3.4. Molecular and Biochemical Analysis of FUCA1 and FUCA2

**Figure 4:**
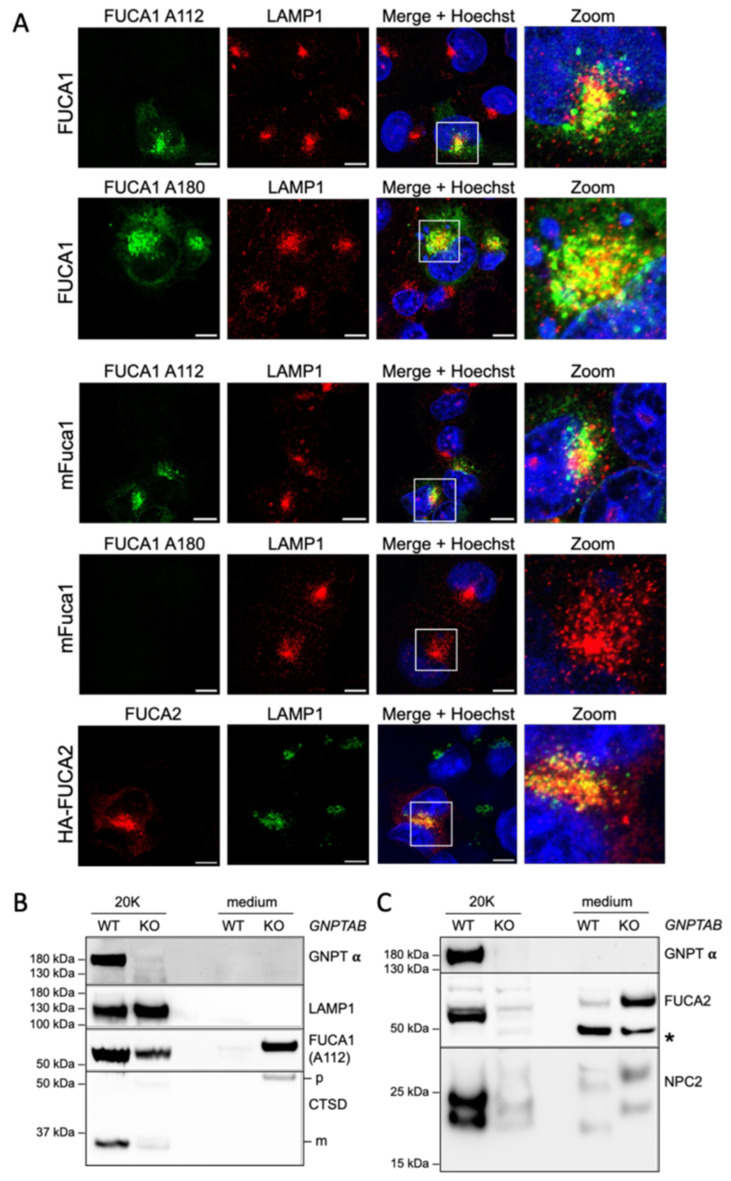
Lysosomal localization of human FUCA1, FUCA2, and mouse (m)Fuca1. (**A**) Transiently expressed untagged FUCA1, mFuca1, or HA-tagged FUCA2 in HT1080 cells were analyzed by double immunofluorescence microscopy using the indicated antibodies. In magnified areas of merged images, yellow indicates the co-localization of fucosidases and lysosomes. Scale bars correspond to 10 µm. (**B**,**C**) Aliquots of extracts from lysosome-enriched fractions (20K) and concentrated media conditioned for 24 h from wild-type (WT) and *GNPTAB*-KO HAP1 cells were analyzed by western blotting for the loss of the GNPT α subunit. The western blots were also probed with antibodies detecting (**B**) the lysosomal membrane protein LAMP1, FUCA1, and cathepsin D (CTSD; p—precursor; m—mature form) and (**C**) FUCA2 and NPC2. * indicates a protein unrelated to FUCA2.

We further explored the suitability of the antibodies A112 and A180 as well as the FUCA2 antibody for application in immunofluorescence microscopy. HT1080 cells were transiently transfected with constructs encoding untagged human (h)FUCA1 or untagged murine (m)Fuca1. Both hFUCA1 and mFuca1 were detectable in vesicular structures with A112, while A180 only recognized the human variant (Figure 4A). We analyzed the subcellular localization of hFUCA1 and mFuca1 by co-staining with the lysosomal membrane marker protein LAMP1. The fluorescence signals for hFUCA1 and mFuca1 partially colocalized with the LAMP1-positive structures. FUCA2 also showed extensive co-localization with the lysosomal marker LAMP1 using the polyclonal FUCA2 antibody after transient transfection, confirming that FUCA2 is a genuine lysosomal protein. However, due to the overexpression conditions, other compartments of the secretory and biosynthetic lysosomal pathway, such as the ER, the Golgi apparatus and the endosomes, might also contain the proteins. It is worth mentioning that both FUCA1 antibodies worked after methanol fixation but not after pFA/Triton X-100 fixation and permeabilization.

The novel FUCA1 antibodies failed to detect endogenous Fuca1 on multiple tissue western blots from wild-type and *Fuca1*-KO mice. However, endogenous FUCA1 and FUCA2 were detected in 20,000× *g* (20K) lysosome-enriched fractions prepared from human wild-type (WT) haploid HAP1 cells (Figure 4B,C). In contrast, HAP1 cells in which the gene encoding the Golgi-resident GlcNAc-1-phosphotransferase α/ß subunit precursor protein (*GNPTAB*) was deleted by CRISPR-Cas9 gene editing showed decreased levels of FUCA1 in the 20K lysosomal fraction, whereas the abundance of other lysosomal enzymes, such as FUCA2 and cathepsin D (CTSD), or of the luminal accessory protein Niemann-Pick type 2C (NPC2), was strongly reduced. The loss of GlcNAc-1-phosphotransferase prevents the modification of luminal lysosomal proteins with M6P targeting signals, resulting in the missorting of a significant proportion of these newly synthesized proteins into the medium rather than correctly directing them to lysosomes. Lysosomal membrane proteins like LAMP1 remained unaffected due to their M6P-independent transport to lysosomes. These results demonstrate that the sorting of both lysosomal α-L-fucosidases, FUCA1 and FUCA2, is strongly M6P-dependent.

**Figure 5:**
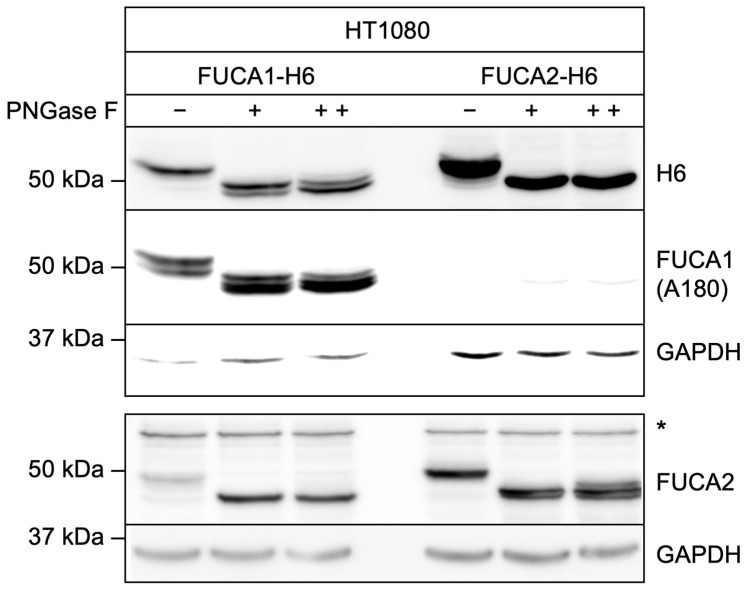
Aliquots of HT1080 cell lysates (40 µg protein) expressing FUCA1-H6 and FUCA2-H6 were incubated in the absence (−) or presence of PNGase F (10 units) for 30 min (+) or for 4 h (++) at 37 °C and analyzed by western blotting using H6-tag, FUCA1 (A180), FUCA2, and GAPDH antibodies. * indicates a protein unrelated to FUCA2.

To examine the N-glycosylation of both fucosidases, HT1080 cell lysates ectopically expressing FUCA1-H6 and FUCA2-H6 were treated with or without PNGase F and analyzed by western blotting (Figure 5). A moderate decrease in the apparent molecular mass from 53/55 kDa to approx. 50 kDa was observed for both fucosidases, indicating the loss of 2–3 *N*-linked oligosaccharides. Furthermore, deglycosylation of FUCA2-H6 did not enable its detection by the A180 antibody. In contrast, the A180 antibody detected three deglycosylated bands for FUCA1-H6, while the H6-specific antibody, which detects the C-terminal H6-tag, only recognized two bands, suggesting that the H6-tag might be partially cleaved off.

### 3.5. Ectopic Expression and Enzymatic Activity of α-L-Fucosidases

**Figure 6:**
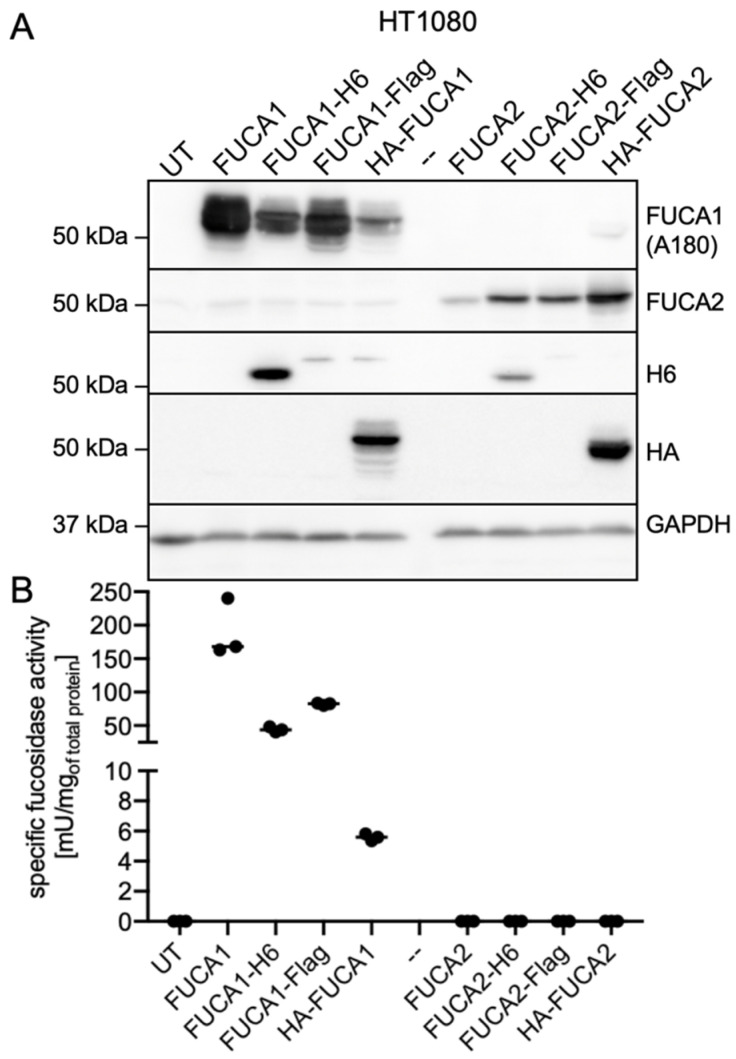
Western blot analysis and fucosidase activities of HT1080 cell lysates transiently transfected with untagged, H6-, Flag-, or HA-tagged *FUCA1* and *FUCA2* constructs. (**A**) Lysates (25 µg total protein) from untransfected (UT) and fucosidase-expressing HT1080 cells were analyzed by western blotting using the indicated antibodies. (**B**) α-L-fucosidase activity of the lysates was determined using the 4-MUF substrate (mean ± SD; *n* = 3).

To examine whether FUCA2 contributes to overall α-L-fucosidase activity, we expressed various human and mouse *FUCA1* and *FUCA2* constructs and analyzed their expression by western blotting as well as their fucosidase activity in lysates of HT1080 cells (Figure 6A,B). The endogenous specific α-L-fucosidase activity of HT1080 cells is 35-fold lower than that of HEK293FT cells (Supplementary Figure S1), which may be advantageous for measuring low FUCA2-mediated fucosidase activity. In addition to the C-terminally H6-tagged FUCA variants, we used different *FUCA1* and *FUCA2* constructs in the same eukaryotic expression vector with a C-terminal Flag-tag, an N-terminal HA-tag, or without any tag (untagged) to prevent possible interference with expression level or enzymatic activity.

In western blots of HT1080 cell lysates, the A180 antibody detected all human FUCA1 derivatives, namely untagged, H6-, Flag-, and HA-tagged FUCA1 (Figure 6A). Densitometric analysis showed that, compared to untagged FUCA1, the expression levels of H6-tagged FUCA1, Flag-tagged FUCA1, and the HA-tagged variant were 58%, 80%, and 29%, respectively. The FUCA2 antibody detected all FUCA2 derivatives, as well as FUCA2 at low endogenous levels in lysates with ectopic FUCA1 expression and in untransfected (UT) HT1080 cells. To verify the expression levels of FUCA1 and FUCA2, we used H6- and HA-tag-specific antibodies, showing that the intensity of the FUCA1-H6 band was significantly higher than that of FUCA2-H6, whereas the HA signal was slightly stronger for HA-FUCA2 than for HA-FUCA1.

Determination of α-L-fucosidase activity using 4-MUF as an artificial substrate revealed the highest α-L-fucosidase activity in HT1080 cells expressing untagged FUCA1 (Figure 6B). H6-tagged FUCA1, Flag-tagged FUCA1 and HA-tagged FUCA1 exhibited 23%, 50% and 3% of the α-L-fucosidase activity of untagged FUCA1, respectively. Considering the intensity of the western blot signals, H6-tagged FUCA1 showed 39% activity, Flag-tagged FUCA1 62% and the HA-tagged variant 11% in comparison to untagged FUCA1. However, no FUCA2-containing HT1080 lysate showed α-L-fucosidase activity exceeding that of untransfected (UT) controls. We also tested untagged and H6-tagged FUCA1 and H6-tagged FUCA2 in HEK293FT cell lysates after ectopic expression but did not detect any increase in FUCA2-mediated fucosidase activity compared to untransfected cells (Supplementary Figure S2).

**Figure 7:**
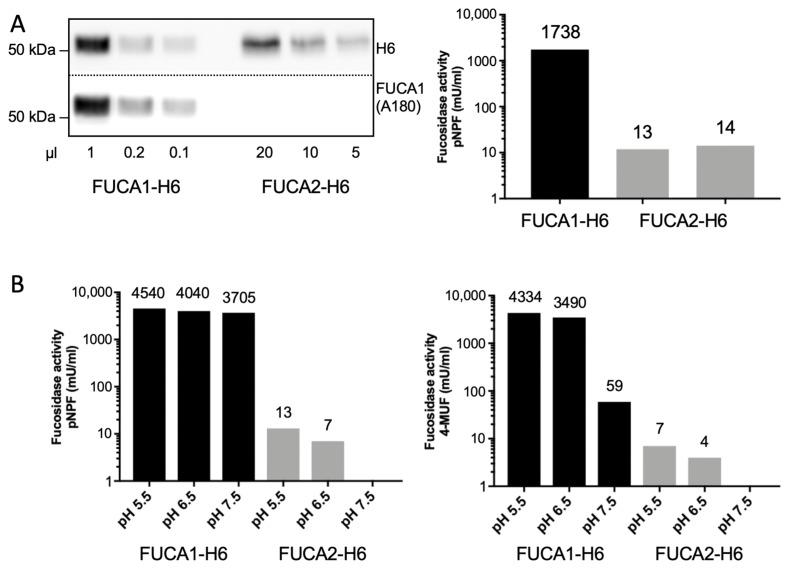
(**A**) Aliquots of affinity-purified FUCA1-H6 and FUCA2-H6 derived from supernatants of stably expressing HT1080 cells were analyzed by western blotting (left panel), and fucosidase activity was determined using pNPF as a pseudo-substrate (right panel). (**B**) Aliquots of the elution fractions (1 µL FUCA1-H6 and 10 µL FUCA2-H6) were used for α-L-fucosidase activity assays using pNPF (left) or 4-MUF (right) as pseudo-substrates at the indicated pH values.

As we failed to detect any FUCA2 activity in lysates upon transient expression, we generated stable FUCA2-H6- and FUCA1-H6-expressing HT1080 cell lines. Both fucosidases were purified simultaneously from approx. 1 L of conditioned media by Ni-NTA-affinity chromatography. The amounts of secreted FUCA1 and FUCA2 in the concentrated and rebuffered 200 µL elution fractions were estimated by western blotting (Figure 7A, left panel), and their purity was assessed by Coomassie-staining (Supplementary Figure S3). Using pNPF as an artificial pseudo-substrate, we failed to detect α-L-fucosidase activity in the FUCA2 sample, in which 20 µL and 10 µL of the FUCA2 elution fraction were used, while 1 µL of the affinity-purified FUCA1 sample showed high fucosidase activity (Figure 7A, right panel).

As FUCA2 was considered to be a second lysosomal fucosidase that might also be responsible for the enzyme activity in serum at neutral pH, we tested the same affinity-purified FUCA1-H6 and FUCA2-H6 fractions at different pH values (Figure 7B). We found that FUCA1 was enzymatically active at pH 5.5, 6.5, and 7.5 with both artificial α-L-fucosidase-specific pseudo-substrates (Figure 7B; pNPF, left panel; and 4-MUF, right panel), while virtually no fucosidase activity was detectable in any of the FUCA2 samples.

### 3.6. Oligomerization of FUCA1 and FUCA2

**Figure 8:**
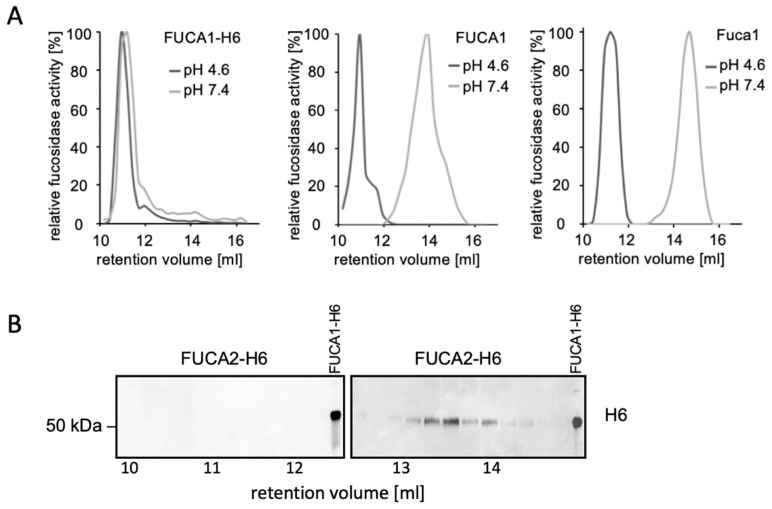
(**A**) Size exclusion chromatography (SEC) using a Superdex 200 Increase column in McIlvaine buffer (pH 4.6) or in PBS (pH 7.4), followed by 4-MUF α-L-fucosidase activity assays of collected fractions (250 µL), was performed using affinity-purified H6-tagged human FUCA1 (left panel), untagged human FUCA1 (middle panel) and native mouse Fuca1 from a liver-derived lysosome-enriched fraction (right panel). (**B**) Affinity-purified H6-tagged human FUCA2 was separated by SEC at pH 4.6 in 250 µL fractions. The monomeric FUCA1/Fuca1 variants (**A**) and FUCA2-H6 (**B**) eluted between the 141 kDa marker protein alcohol dehydrogenase (at approx. 12.5 mL) and the 43 kDa marker ovalbumin (at approx. 15 mL).

Previously, a pH-dependent tetramerization or even higher forms of oligomerization of FUCA1 have been discussed [38,39], and very recently, the homotetrameric structure of human recombinant FUCA1 was shown by cryo-EM [14]. The capability of FUCA2 to oligomerize has not been investigated so far. We purified untagged FUCA1 using strong ion-exchange chromatography (SCX), FUCA1-H6, and FUCA2-H6 by Ni-NTA chromatography, and additionally obtained native mouse Fuca1 from lysosome-enriched fractions (so-called *tritosomes*) from mouse liver. These different purified FUCA1/Fuca1 forms, as well as the affinity-purified FUCA2-H6, were analyzed by size exclusion chromatography (SEC) at lysosomal and neutral pH. For the different FUCA1 derivatives, collected fractions were tested for α-L-fucosidase activity (Figure 8A), while FUCA2-H6 was detected by H6-tag western blotting due to the lack of α-L-fucosidase activity (Figure 8B). Under acidic conditions, recombinant FUCA1-H6 (Figure 8A, left panel) as well as untagged FUCA1 (Figure 8A, middle panel) eluted in an oligomeric form, with apparent molecular masses of 200–300 kDa according to the standard protein calibration (see Supplementary Figure S4). Moreover, fucosidase activity derived from wild-type mouse liver *tritosomes* was detected at a comparable retention volume at acidic pH, also demonstrating an oligomeric Fuca1 in mouse liver lysosomes (Figure 8A, right panel). Under neutral conditions (pH 7.4), we observed differences between the FUCA1-H6 and the untagged forms of FUCA1 (middle panel), and Fuca1 (right panel), which eluted only as approx. 50 kDa monomers. In contrast, FUCA1-H6 appeared as an oligomer at both pH values (left panel). Affinity-purified H6-tagged FUCA2 was accordingly separated by SEC and was detectable in fractions corresponding to the monomeric form (Figure 8B). In summary, these data suggest a pH-dependent oligomerization of FUCA1/ Fuca1, whereas FUCA2 shows no oligomerization.

## 4. Discussion

In humans, the lysosomal degradation of fucosylated glycoconjugates depends on α-L-fucosidase activity, which is provided by the thoroughly characterized acidic tissue α-L-fucosidase (FUCA1) that is also involved in, for example, cancer progression and antigen processing [40,41,42]. The contribution of a second α-L-fucosidase, namely FUCA2, to lysosomal bulk de-fucosylation remains under discussion due to very limited experimental evidence for its enzymatic capability. The conserved overall structure of both human proteins, as well as the conserved position of critical amino acids within the catalytic domain of both proteins, such as the catalytic nucleophile (FUCA1 D230 vs. FUCA2 D228), the proposed catalytic acid/ base (FUCA1 D281 vs. FUCA2 D279) and the stabilizing glutamate (FUCA1 E294 vs. FUCA2 E292), principally argue for a fucosidase activity of FUCA2.

To enable a comparative analysis of both fucosidases, we generated and evaluated two FUCA1-specific monoclonal antibodies and also applied a commercially available FUCA2-specific antibody. Using these antibodies, we detected FUCA1 and FUCA2 after ectopic expression in lysates of various cell lines, including HT1080, HeLa, and HEK293FT cells. Moreover, we were able to visualize FUCA1 and FUCA2 in immunoblots of lysates from haploid HAP1 WT cells, as well as from HeLa, HEK293FT, SK-MEL-28, and SK-MEL-30 cells, at endogenous levels. As expected for soluble lysosomal proteins sorted in an M6P-dependent manner, HAP1 cells deficient in the M6P-generating GlcNAc-1-phosphotransferase (GNPT) largely secreted both fucosidases. In this line of evidence, PNGase F treatment confirmed the N-glycosylation of both fucosidases, and immunofluorescence microscopy using the fucosidase antibodies confirmed the lysosomal localization of FUCA1 and FUCA2. Many studies have suggested a lysosomal localization rather than secretion for FUCA2, as it has been consistently identified in subproteomic analyses of mannose 6-phosphorylated (M6P) glycoproteins [23,26,27,43,44]. Overall, these results demonstrate that FUCA2 is a bona fide lysosomal glycoprotein that is transported into the lysosomal compartment in an M6P-dependent manner.

To further evaluate whether FUCA2 might contribute to overall fucosidase activity, we cloned and expressed untagged and various tagged FUCA2 derivatives in HT1080 cells, which are characterized by the lowest endogenous fucosidase activity, and compared them with FUCA1 variants with regard to α-L-fucosidase activities using the specific artificial substrates 4-MUF and pNPF. In particular, pNPF seems to be the most appropriate substrate, as demonstrated by Perna et al., who analyzed 96 GH29 fucosidases from different species for their substrate specificity [45]. No FUCA2-mediated activity was detected under any of the conditions tested in our study, even though we used different FUCA2 derivatives (tagged, untagged, human or mouse) from different sources (cell lysates or affinity-purified FUCA2) under various assay conditions (pH, buffer, and pseudo-substrates). Consequently, it remains questionable whether FUCA2 is an active fucosidase.

So far, experimental data on direct FUCA2-mediated fucosidase activity are limited to a single approach, in which FUCA2 secretion and activity were shown to be necessary for *Helicobacter pylori* adhesion to host cells via bacterial Lewis X structures [25]. Since FUCA1-deficient fucosidosis patients exhibit, in addition to the accumulation of many fucosylated glycoconjugates, massive storage of Lewis X and H antigen-glycolipids in tissues and urine [15], it is rather unlikely that FUCA2 has a hydrolytic activity towards these substrates. Furthermore, most fucosidosis patients were found to have excessive accumulation of two other Lewis antigens, namely α(1,4)-mono-fucosylated Lewis a and α(1,2)-/α(1,4)-bi-fucosylated Lewis b. The combined occurrence of both Lewis antigens is very rare in the normal population (0% to <1%) and supports a general loss of fucosidase activity [6,46].

Considering that lysosomal FUCA1 has been shown to catalyze the removal of all kinds of α-glycosidically linked fucosyl residues from a broad spectrum of natural substrates as well as synthetic pseudo-substrates [47,48], it remains questionable which (natural) substrates could be left for degradation by the FUCA2 enzyme. Both established *Fuca1*-based fucosidosis mouse models completely lacked any fucosidase activity and exhibited a massive storage of various fucosylated glycoconjugates in numerous tissues and urine [16,17,36], which argues against a functional enzymatic redundancy of both fucosidases. We also determined overall fucosidase activity at pH 7.4 in plasma and serum of our Fuca1-deficient mice and failed to detect any residual fucosidase activity, indicating that Fuca1 represents the only active fucosidase, at least in mice [49]. Moreover, the homozygous knockout of *Fuca2* in mice, in which exons 1 and 2 were targeted, resulted in a normal phenotype without signs of pathogenic lysosomal accumulation of (fucosylated) substrates [50]. Finally, no clinical case with lysosomal storage of predominantly fucosylated glycoconjugates has yet been reported that is attributable to defects other than FUCA1.

*Fuca2* transcript levels were not consistently elevated in our established *Fuca1* knockout mouse model, which might be expected if Fuca2 compensated for the lack of Fuca1 activity. Interestingly, the transcript levels, protein levels, and the enzymatic activities of several lysosomal hydrolases, such as ß-hexosaminidase (HexB), lysosomal α-mannosidase (Man2b1), lysosomal α-glucosidase (GAA), glucosylceramidase (GBA), and cathepsin D (CtsD), were increased in *Fuca1*-knockout mice [16,17,36]. This most likely reflects the enlarged endosomal-lysosomal compartment and lysosomal stress, followed by transcription factor EB (TFEB)-mediated activation of target genes, such as *CtsD*, *GAA*, *GBA*, *HexB*, and also *FUCA2* [51,52]. Therefore, it seems surprising that *Fuca2* transcript levels are not transcriptionally influenced upon Fuca1 deficiency.

The recent structural analysis of FUCA1 by cryo-EM revealed its homotetrameric structure and concretized previous studies, in which dimeric, tetrameric and also hexameric forms were reported [5,13,38]. The pH-dependent switch between monomer and oligomer, as observed here for human FUCA1 and mouse Fuca1 from liver *tritosomes*, was previously described for FUCA1 from human placenta [38] and closely resembles the behavior of lysosomal arylsulfatase A (ARSA), which forms dimers before reaching the Golgi apparatus and further oligomerizes to octamers at lysosomal pH [53].

In conclusion, none of our experimental data support the concept of FUCA2-mediated fucosidase activity, leaving the physiological function of FUCA2 unresolved. Future comparative studies using both FUCA1- and FUCA2-deficient cells are needed to analyze fucose-containing *glycoproteomes* and *glycolipidomes*, thereby enabling the characterization of enzymatic or non-enzymatic functions of FUCA2. These studies are a prerequisite for a better understanding of the role of increased *FUCA2* expression in many tumor types, which is associated with poor survival rates [54].

## Data Availability

The original contributions presented in this study are included in the article/Supplementary Materials. Further inquiries can be directed to the corresponding author.

## Supplementary Materials

The following supporting information can be downloaded at: https://www.mdpi.com/article/10.3390/cells14171355/s1, Figure S1: Lysates ofuntransfected (UT) HT1080 cells, HeLa cells and HEK293FT cells were tested for specific α-Lfucosidase activity using the 4-MUF substrate (mean ± SD; n = 3). Figure S2: Lysates of untransfected (UT) as well as FUCA1-H6 and FUCA2-H6 expressing HEK293 cells were analyzed by western blotting using the indicated antibodies and were tested for specific α-L-fucosidase activity using the 4-MUF substrate (mean ± SD; n = 3). Figure S3: Aliquots of affnity purified FUCA1-H6 and FUCA2-H6 were separated by SDS-PAGE and detected by Blue silver colloidal Coomassie G-250 staining procedure (Candiano et al. (2004) Electrophoresis 25(9):1327-1333). Figure S4: Standard calibration proteins were separated by size exclusion chromatography (SEC) using a Superdex 200 10/300 GL column (Cytiva, Freiburg, Germany) for molecular mass calculation at pH 4.6 (A) and at pH 7.4 (B): thyroglobulin 660 kDa; ferritin 440 kDa; beta-amylase 215 kDa; alcohol dehydrogenase 140 kDa; ovalbumin 45 kDa; cytochrome c 12.5 kDa; aprotinin 6.5 kDa.

## Abbreviations

The following abbreviations are used in this manuscript:

FUCA1	tissue α-L-fucosidase 1
FUCA2	plasma α-L-fucosidase 2
qPCR	quantitative polymerase chain reaction
4-MUF	4-methylumbelliferyl-α-L-fucopyranoside
pNPF	p-nitrophenyl-α-L-fucopyranoside
BSA	bovine serum albumin
DMEM	Dulbecco’s Modified Eagle’s Medium
MEF	mouse embryonic fibroblasts
Ct	cycle threshold
RT	room temperature
