# Supplementary material for: Deciphering α-L-Fucosidase Activity Contribution in Human and Mouse: Tissue α-L-Fucosidase FUCA1 Meets Plasma α-L-Fucosidase FUCA2
Source: Cells. 2025 Aug 30;14(17):1355. doi: 10.3390/cells14171355 (PMC12427801; doi:10.3390/cells14171355)

Article

Deciphering α-L-fucosidase activity contribution in human and mouse: Tissue α-L-fucosidase FUCA1 meets Plasma α-L-fucosidase FUCA2

Hannah Bäumges[1], Svenja Jelinek[1], Heike Lange[1], Sandra Markmann[2], Emanuela Capriotti[2], Jan Anwar Häusser[1], Mai-Britt Ilse[1], Thomas Braulke[2], and Torben Lübke[1]

Suppl. Figures S1 - S4

Suppl. Figure S1: Lysates of untransfected (UT) HT1080 cells, HeLa cells and HEK293FT cells were tested for specific α-L-fucosidase activity using the 4-MUF substrate (mean ± SD; n=3).

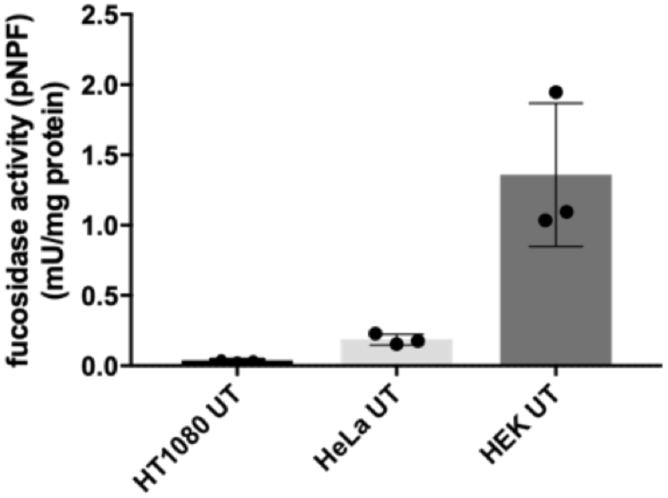

Suppl. Figure S2: Lysates of untransfected (UT) as well as FUCA1-H6 and FUCA2-H6 expressing HEK293 cells were analyzed by western blotting using the indicated antibodies and were tested for specific α-L-fucosidase activity using the 4-MUF substrate (mean ± SD; n=3).

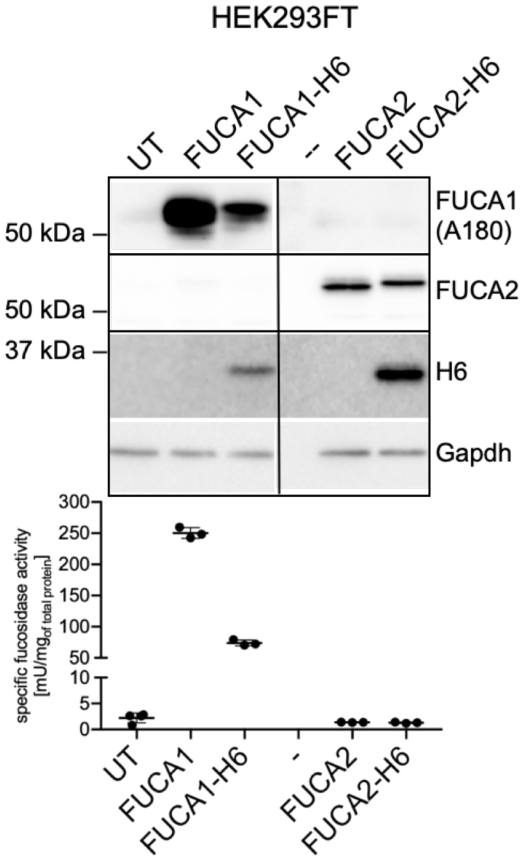

Suppl. Figure S3: Aliquots of affnity purified FUCA1-H6 and FUCA2-H6 were separated by SDS-PAGE and detected by Blue silver colloidal Coomassie G-250 staining procedure (Candiano et al. (2004) *Electrophoresis* 25(9):1327-1333).

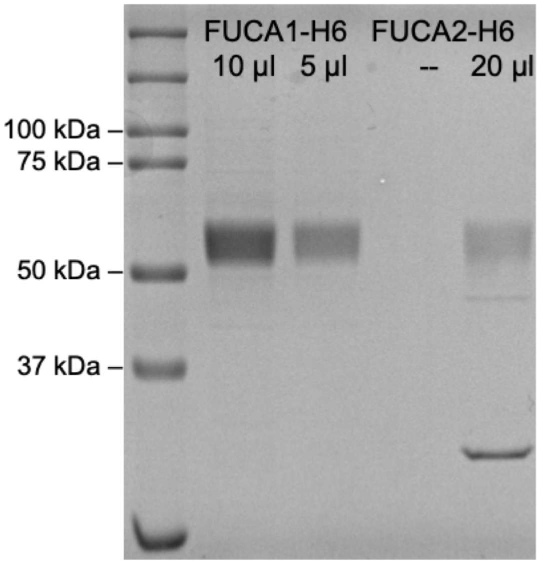

Suppl. Figure S4: Standard calibration proteins were separated by size exclusion chromatography (SEC) using a Superdex 200 10/300 GL column (Cytiva, Freiburg, Germany) for molecular mass calculation at pH 4.6 **(A)** and at pH 7.4 **(B)**: thyroglobulin 660 kDa; ferritin 440 kDa; beta-amylase 215 kDa; alcohol dehydrogenase 140 kDa; ovalbumin 45 kDa; cytochrome c 12.5 kDa; aprotinin 6.5 kDa.

A

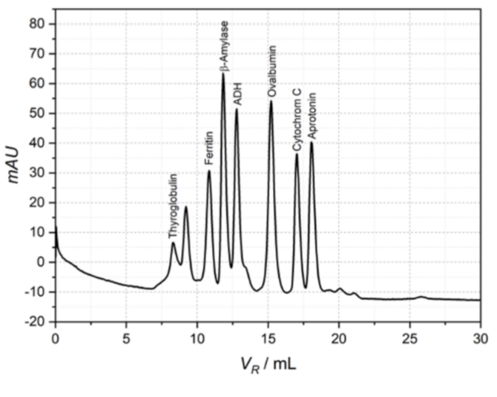

B

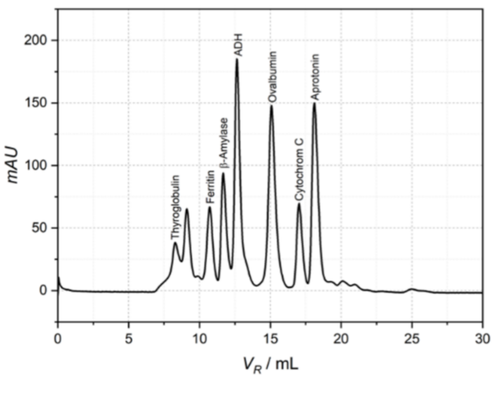

Supplement: Supplementary file 1 [file cells-14-01355-s001.zip › cells-3613727-supplementary.pdf]
